# Toward the Web of Industrial Things: A Publish-Subscribe Oriented Architecture for Data and Power Management

**DOI:** 10.3390/s22134882

**Published:** 2022-06-28

**Authors:** Claudio Bartoli, Michele Bonanni, Francesco Chiti, Laura Pierucci, Alessandro Cidronali, Giovanni Collodi, Stefano Maddio

**Affiliations:** Department of Information Engineering, University of Florence, 50139 Firenze, Italy; claudio.bartoli@stud.unifi.it (C.B.); michele.bonanni@unifi.it (M.B.); laura.pierucci@unifi.it (L.P.); alessandro.cidronali@unifi.it (A.C.); giovanni.collodi@unifi.it (G.C.); stefano.maddio@unifi.it (S.M.)

**Keywords:** industrial wireless sensors and actuators networks, web of industrial things, mqtt protocol, wireless power transfer

## Abstract

The foundation of an energy sustainable Web of Industrial Things (WoIT) is facing several open issues due to the constraints imposed by the involved devices, the technological heterogeneity and the complex interactions and, hence, communications patterns. Towards this goal, in this paper, a general framework inspired by the Publish-Subscribe principle have been proposed, in order to jointly optimize the service requirements and the network availability. In particular, in this paper we focus on a holistic design with the objective to manage power budget distribution, in order to support applications that extend the basic publish-and-subscribe scheme.The involved WoIT nodes functionalities, interfaces and hardware architectures have been designed, with a special focus on control protocols. The introduced integrated solution has been validated in scenarios minimising and possibly balancing the power consumption. The achieved results show an average improvement of 45% for the communications performance with the wireless power management.

## 1. Introduction

The so called *Internet* of Things (IoT) is widely considered as one of the most disruptive paradigms accelerating the transition to a novel technological and societal era [1]. An IoT domain can connect a large number of resource-constrained devices (namely the *things*)with heterogeneous characteristics, in terms of category, functionality, manufacturer up to the adopted communications protocols, then requiring novel and flexible network architectures to meet a variety of new applications and users’s requirements in real-time [2].

In particular, IoT ecosystem could be considered an extremely relevant and promising component of the future industrial processes, that are alternatively indicated as the Industrial Internet or the Industry 4.0. Industry 4.0 is converting traditional industrial automation and control systems into cyber-physical manufacturing systems by integrating Information Technology (IT) and Operational Technology (OT). In this vision, industrial wireless sensors and actuators networks (IWSANs), may contribute to overcame the drawbacks and constraints of the wired communications technologies usually implemented in industrial scenarios. IWSANs decrease the installations and maintenance costs since they do not requires to deploy or protect cables even in hostile industrial areas. Moreover, they allow easy reconfiguration and, possibly, the support for devices mobility, and, consequently, the control of robot. However, IWSANs and in general the Industrial loT (IIoT) systems are becoming more complex with growing scales, which leads to a number of significant challenges that need to be addressed, such as increasing energy consumption [3,4].

In order to provide a general purpose IIoT platform to easily set up a distributed application, it has been investigated the adoption of the so called Web of Things (WoT) approach [5]. The information exchange occurring as an WoT domain interacts with the physical world is characterised by specific features, mainly dictated by the *constrained* devices which suffer of limited processing power, network bandwidth, and intermittent connectivity. In addition, communications are sporadic and often event-triggered updates, with continuous multiple data flows [6]. To address these limitations, the Constrained Application Protocol (CoAP) has been firstly proposed to adapt HTTP for constrained devices and to enable more advanced interaction patterns w.r.t. HTTP, e.g., the support for multicast [7]. Another promising candidate is represented by the Message Queue Telemetry Transport (MQTT) protocol designed for monitoring applications. It is based on the publish-and-subscribe paradigm, by which Publishers (e.g., sensors) transmit data messages to a Broker, which in turn delivers such messages to interested entities, called Subscribers [8]. This flexible approach places complexity in the Broker. Furthermore, MQTT defines a lightweight header format and requires a small code footprint. Recently, the industrial Open Platform Communications Unified Architecture (OPCUA) adopted the publish/subscriber model in its release 14, and the MQTT messages are used for the broker-based case. Moreover, a version of this protocol, called MQTT for Sensor Networks (MQTT-SN), has been specifically designed to face the constraints of a typical IoT domain with suitable architecture, interfaces and components [9].

In a smart manufacturing environment endowed with IWSANs, actuators and sensors are usually battery powered. This requires a specific maintenance cycle which can became a daunting task with hundreds of devices installed in harsh and hardly accessible locations. As a consequence, an energy-saving approach for IWSANs should be considered to improve the networks lifetime. In principle, two possible methodologies for supplying energy to devices can be considered: Energy Harvesting (EH) and Wireless Power Transfer (WPT) [10].

Both the approaches represent promising methodology but differ conceptually. EH converts every possible available energy source existing in a specific environment into power to supply the device. An optimum implementation of this approach requires an accurate identification of both spatial and temporal energy distribution in the operation enviroment in order to power up the devices in accordance with the functional requirements [11]. As a consequence, the knowledge of these parameters drives the overall system design in term of performance, complexity and functionalities [12]. Recently an EH approach based on the use of renewable energy sources has benn proposed to achieve long-term and self-sustainable operations for nodes. This traslates in prolonging the lifetime of IoT network especially if operating in extreme environments where the replacement of batteries can be difficult. The latter represents a great improvements with respect of applications where a mobile charger is usually employed to provide the energy replenishment through energy downlink collecting surplus power from energy-rich nodes and transfer it to energy-deficient nodes. An EH system consists of two processes: (1) energy harvesting and (2) data transmission. Since data communications modify the nodes energy, an optimal control of data and related energy flows represents a critical issue for effective communications.

On the other hand, WPT supports both battery-less and backup-battery approach, giving a higher degree of freedom for the system designers [13]. In particular, WPT enables the energy supply with a direct device activation, or the device battery recharge, respectively. The second approach relies on an infrastructure dedicated to the energy transfer embodied in the communications topology, requesting specific routing strategies to optimise both aspects [14].

The present work is focused on an overall system architecture design for application over an IIoT scenarios. In particular, we consider that industrial devices (i.e., sensor and actuators) are expected to act as a group to perform homogeneous operations in terms of manufacturing and quality assessment. Then they can be logically clustered and the possible interactions among clusters needs to be optimised mostly under energetic constraints which affects their availability and life-time. In order to establish intra- and inter-cluster(s) communications, we resorted to a WoT protocol, in particular, following the publish-and-subscribe pattern, according to the MQTT-SN framework. This allows to set-up extremely flexible end -to-end (e2e) sessions; to this purpose, we derived a solution that allows the joint management of the data flows and power budget management. Specifically, the aim is to evaluate e2e paths among Publisher(s) and Subscriber(s) minimising the power consumption. Accordingly, we characterised the proposed architectures and interfaces, further extending this approach by taking into account also the presence of a subnetwork to handle the WPT procedure, that is aimed to dynamically distribute the available power to that nodes involved in communications. The involved functional modules and the device level architectures have been described and harmonised within the overall system.

Summarising, the main contribution of this paper consists in:

- an high-level architecture design, integrating a typical IIoT scenario on a publish-subscribe oriented messaging framework through proper interfaces and protocols
- individual module modelling and design, with a specific focus on the power supplying process, both on a component and procedure point of view.
- development of an ad hoc simulation framework including different approaches performed over realistic scenarios.

The paper structure is at it follows: after introducing the specific features, the related literature and the open issue of IIoT domain in Section 1, the reference system model is presented in Section 2, in terms of both the general architecture and specific communications and control protocols, with a characterisation of hardware components. The overall performance is investigated in Section 3 for different device deployments, pointing out a remarkable gain in terms of network life-time. Finally, the conclusions are drawn in Section 4.

## 2. System Model

### 2.1. Proposed Integrated Architecture

The proposed solution aims at optimising the overall energy consumption of a clustered IIoT scenario, leveraging the WPT concept and, consequently, maximising the services availability and system durability to support typical industrial applications. Specifically, this ha been accomplished by introducing the following elements: (i) an Energy Supplier (ES) node, which is in charge of handling the WPT procedure and possibly acting as a communication gateway toward external networks, (ii) a Rechargeable Node (RN) which represent a IIoT able to be properly recharged thank to the harvester block, and (iii) the Power Transfer Manager (PTM) which optimise the power transfer by enforcing a suitable strategy.

**Figure 1:**
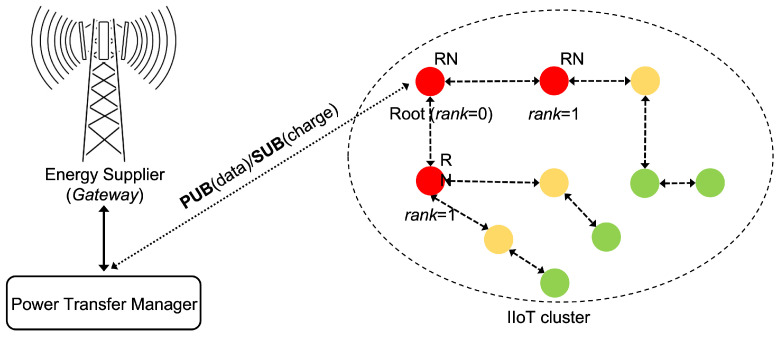
High level architecture comprising the Energy Supplier node and the Power Transfer Manager which jointly handle the WPT procedure for an IIoT cluster arranged in a tree-wise topology, in the presence of Rechargeable Nodes.

In Figure 1, the high level architecture is depicted, where each IIoT cluster is arranged in a tree-wise topology, according to the Pv6 Routing Protocol for Low-Power and Lossy Networks (RPL) [15], which has been modified to select the root and lower *rank* nodes among the RNs. It is worth noting that the power consumption is mainly due to packet transmission and reception; as a consequence in each cluster the lower rank nodes are mainly affected by power constrains. Typically, the root node (rank = 0) that forwarda all the messages from/to all the other nodes is likely to firstly deplete their battery. For this reason, it is fundamental that the lower rank nodes were possibly placed closer to ES in order to be recharged when needed to handle an amount of messages greater than the other nodes. According to our proposal, PTM leveraging on the publish-and-subscribe message exchanging, can monitor the residual battery level for the devices that subscribed to the WPT services (i.e., RNs), schedule a WPT cycle on a weighted round robin basis for each cluster, and properly coordinating the ES circuitry. In order to implement the proposed system architecture two specific configurations for both the ES and RN have been conceived, as pointed out in the following.

### 2.2. Energy Supplier Node Architecture

The ES node has been conceived to act primarily as a WPT device for supplying energy to the WSN IoT devices and as a communication gateway. The proposed architecture is based on a dual frequency eight flat face Switched Beam Antenna (SBA) operating at both 2.45 GHz and 5.8 GHz in circular polarization (CP). CP has been implemented in order to provide robustness in both indoor and outdoor scenarios, since it can contrast multipath (indoor) and Faraday rotation (outdoor). Furthermore, CP is not affected by the terminals relative orientation, this avoiding antenna polarisation mismatching in the power link, that consequently could nullify WPT advantages. However, for our purpose, a single antenna cannot be an effective radiator, since the transmitting node, in order to achieve high power transfer efficiency, should focus the transmitted power to the receiver through a narrow beamwidth. In a dynamic scenario, the relative position of transmitter and receiver are not a priori known. Hence, an adequately narrow beam should be dynamically steered to adaptively direct all the available power towards the target receiver in far or near field.

**Figure 2:**
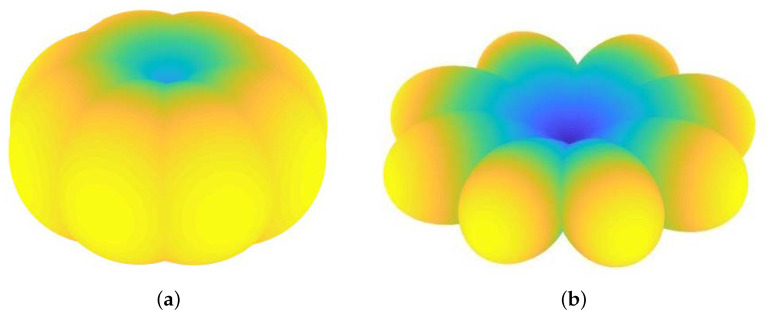
3D SBA composite radiation patterns of the Energy Supplier node at 2.45 GHz (**a**) and 5.2 GHz (**b**).

SBAs are cost-effective radiative systems capable to cover an omnidirectional domain as a whole, but able to selectively switch-on the trasmission toward specific directions, hence operating as spatial filter according to the Space Division Multiple Access (SDMA) paradigm. SBAs are typically controlled by means of a multiplexer, thus it is simpler and more cost-effective than electronically steered array. The better solution for both communication and WPT purposes is to adopt a dual band, where each operation is carried out at different frequency. In view of these considerations, we design and simulate a Dual Band Switched Beam Antenna (DBSBA). The basic antenna element considered in this paper is based on an approach already demonstrated in developing a dual band antenna for indoor communication [16]. The latter being designed for dual band WiFi operation working 2.45 GHz and 5.2 GHz [17]. The SBA final arrangement is obtained introducing the octagonal shape, which is sufficient to cover the entire 2D plane considering the performance of the antenna. Figure 2 shows the 3D simulated radiation pattern for the SBA at the considered frequencies.

**Figure 3:**
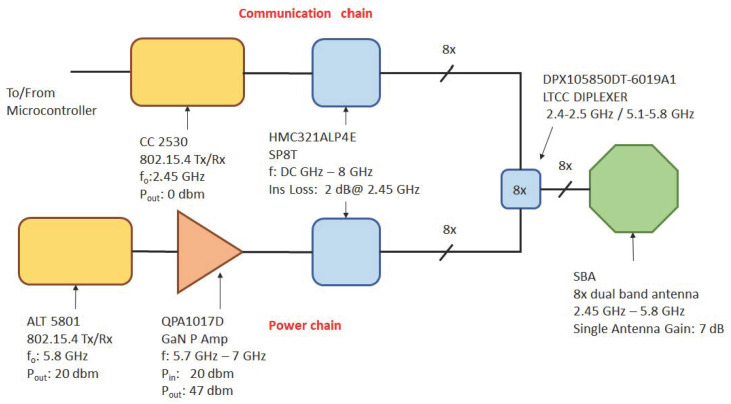
Energy Supplier node block diagram.

The block diagram of the ES proposed architecture is shown in Figure 3, where it is pointed the presence of both the communication and power transfer chains operating at the two frequencies before introduced. The above presented solution defined for the ES is capable to supply power to RNs and particularly to the root node, as well as to communicate with the cluster. The two chains interact with the SBA through eight parallel double frequencies Diplexer driven by two single-pole eight-trough SP8T switch. These ones are connected, respectively, in the 2.45 GHz communication chain to a half-duplex transceiver, while in the 5.8 GHz one, first to a 27 dB gain Power Amplifier, with an output power of about 47 dBm, then to a 5.8 GHz transceiver used to generate a CW signal with an output power level of 20 dBm. In order to demonstrate the feasibility of such architecture, the performance of each functional block corresponds to a commercially available components, as illustrated in Figure 3, where the characteristics of the block match with its specific datasheet.

As a result, ES has the ability to supply power to a target node, as well as communicate with it, while the two SP8T switch both the power and the TX/RX signals to the corresponding active lobe. In this way, the active lobe of the SBA rotates and depict the 3D radiation patterns as in Figure 2. The switching time of SP8T, which correspond to 150 nsec, does not introduce significant latency in both power transmission and communication, due to his inherently negligible impact on the typical on/off time interval.

### 2.3. Rechargeable Node Architecture

**Figure 4:**
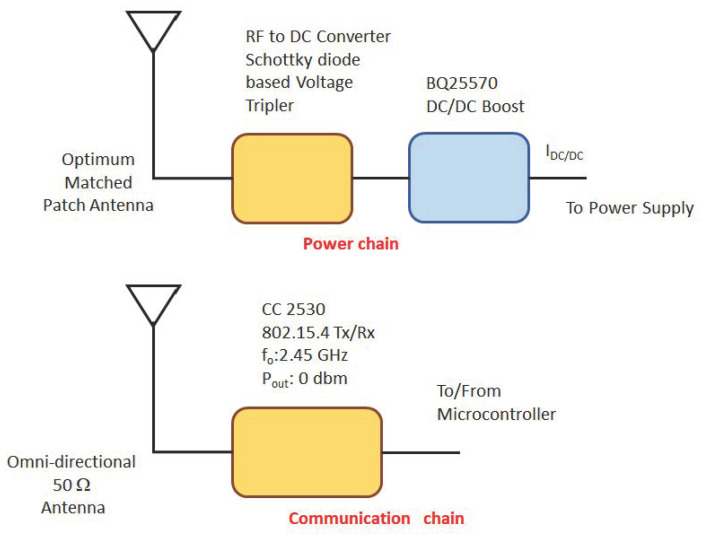
Rechargeable Node power and Tx/Rx chaines.

In order to properly characterize the proposed WPT based approach, also the RN architecture has been defined. The related block diagram is described Figure 4. Each RN is equipped with a 2.45 GHz communication chain adopting an omnidirectional antenna and a 5.8 GHz harvester chain using a linear directional patch antenna designed according to a non canonical optimum impedance matching. The 5.8 GHz RF power, supplied by the ES during the WPT task, is collected by the RN and turned into DC power supply. The energy conversion within the harvesting chain is obtained by means of a solution proposed in [18].

The latter architecture, which is described in Figure 4, makes use of an 5.8 GHz linear optimum matched patch antenna connected to a voltage triplier and followed by a commercial DC-DC Boost BQ25570. The optimum matching impedance is obtained by means of a source-pull optimization carried out on Keysight Advance Design System (ADS) integrating the diode model and a custom BQ25570 behavioral model. The optimum impedance being the one maximizing the BQ25570 input current and, consequently, the power transfer performance.

In the proposed solution, the antenna shows directly the optimum impedance to the voltage triplier minimizing the insertion loss and maximizing the DC-DC boost output current IDC. Taking into account a configuration as the one illustrated in [19], and considering for the present node the same output current parameter, a planar distribution of the current is made available to the node by the WPT block.

### 2.4. WPT Modelling

To completely characterise the WPT process, a model describing the current which is effectively available to RN has been developed and implemented by taking into account the results obtained from electromagnetic and ADS simulations, as well as resorting to a set of measurement illustrated in [18].

**Figure 5:**
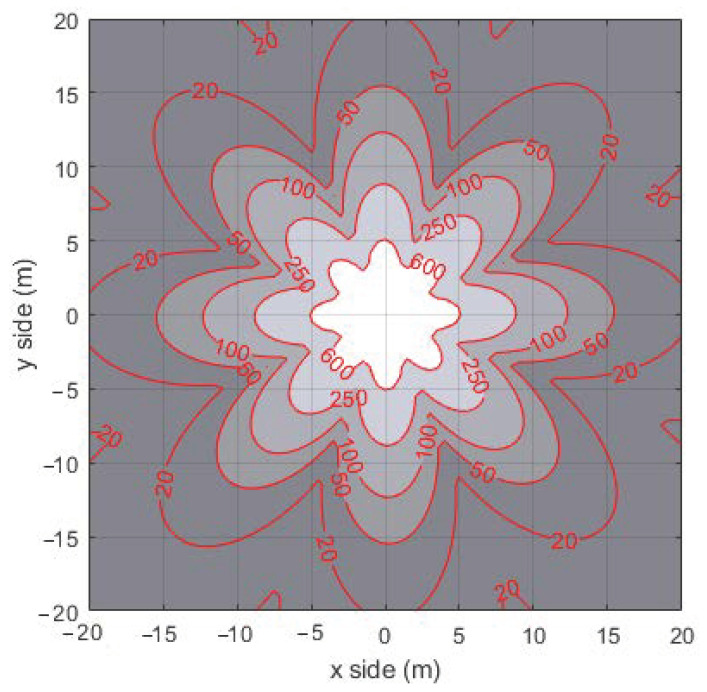
Planar distribution of RF power converted in DC (I_DC_ in uA).

The overall final model consists in the 2D spatial current distribution illustrated in the Figure 5. The procedure followed to obtain it starts with the simulated 5.8 GHz radiation pattern of the SBA Figure 2. As described in Section 2.2, it has been evaluated through an EM simulation that consider the total SBA antenna as a whole. This radiation pattern has been translated in a 2D RF power distribution that depends from the planar (*x*,*y*) coordinates. The current distribution has been calculated by considering the RF to DC conversion efficiency of the RN and taking into account the ES antenna gain. In particular, the RF to DC conversion performances have been calculated basing on the measurements illustrated in [18], that correlate the RF power available at the input of the voltage triplier to the DC current. The inclusion of the ES antenna gain into the SBA 2D RF power planar distribution maps it in the 2D RF planar distribution at the input of the RN voltage triplier. The results is the 2D current planar distribution are illustrated in Figure 5.

Finally, the derived current distribution has been included in a Matlab framework in order to describe the energy made available from the ES to each RN as a function of its 2D position within the described area [17,20].

### 2.5. Power Transfer Manager

The PTM application is mainly focused on the optimisation of the power transfer process, and, consequently, the battery recharging. As previously introduced, this technique allows the GW nodes to receive the energy from ES; in particular they are equipped with ad-hoc circuitry to recharge their battery. In order to avoid energy waste and optimise the recharge process, a synchronisation procedure between the ES and the specific cluster is necessary. Each IIoT cluster is arranged in a tree topology where a unique root node that is interconnected with outer networks, and, during a proper time interval, all the GW nodes belonging to it can receive energy from ES. This is accomplished by PTA which schedules the cluster charging operations. Every GW node sends to PTA its battery level relying on the on the publish-and-subscribe communications framework, together with its Cluster ID and the current tree level (i.e., rank); this information is used to update the network view and perform the cluster selection. The selection is based on the weighted mean residual battery level of an IIoT cluster, where the weighting factor is inversely proportional to the node rank. Once the cluster has been identified, PTA notifies to the intended cluster nodes the starting of the recharging process by sending a specific publish message.

## 3. Performance Analysis

The performance of the proposed approach has been evaluated by means of numerical simulations conducted in the MATLAB framework. Specifically, the simulated nodes are based on IEEE 802.15.4 standard and are divided in sensors and actuators. A sensor is capable to observe homogeneous physical parameters, while an actuator can gather data from other devices and perform specific tasks. The simulated IWSAN nodes have been modelled considering the data sheet of a commercial sensor node as indicated in Table 1. Specifically, we considered a initial battery level of 2500 mAh, corresponding to 2 AAA batteries, a keep alive consumption of 1 μA, a radio transmission consumption of 17 mA and finally a reception consumption of 20 mA.

The transmission power mainly depends on the transmission duration, which is correlated to the packet size and to the adopted data rate. The former was set to 50 bytes, while the latter was set equal to the maximum rate allowed in the IEEE 802.15.4 standard, that is 250 kbps. Finally, the overall node consumption has been completed adding a fixed value of 15 mA for sensing operations. The CPU consumption has not been considered because it is negligible with respect to the transmission and reception power consumption. The WPT has been modeled considering the result shown in Figure 5 and a constant recharging period of 5 s. This value has been evaluated considering an information publishing rate equal to 2 packet/s and a maximum tolerable packet loss of 10 packets. Moreover, we adopted the MQTT-SN standard as the application protocol since it represents a solution suited for IIoT in the presence of resources constrained devices, but we further embodied also the WPT module in the design of the proposed WPT-MQTT-SN. For the sake of comparison, we have considered the CoAP protocol performance.

The network has been partitioned in logical clusters, each managed by its own CH that specifically handles the traffic from or to the ES, where the PTA is executed. The overall system performance has been evaluated in terms of network lifetime, which indicated the network duration. In particular, we adopted a strict definition, that is the time elapsed since the first node in the network depletes its battery. Results have been obtained considering a initial battery level of 150 mAh. Furthermore, the proposed system has been tested over three different nodes deployment: (i) Random Mesh, (ii) Two-Tier, and (iii) Unbalanced Random Mesh and, for each of them we evaluated how the data transmission frequency impacts on the WPT procedure. Finally, the overall number of devices has been assumed to be equal to 40, in compliance with most common IIoT scenarios.

**Figure 6:**
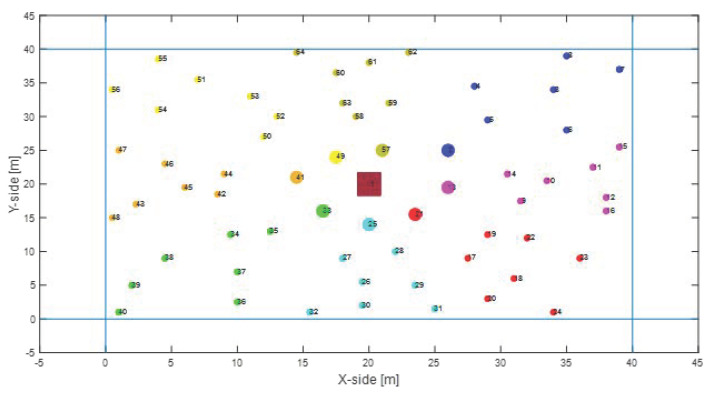
Random Mesh nodes deployment, where clusters are highlighted in different colours.

**Figure 7:**
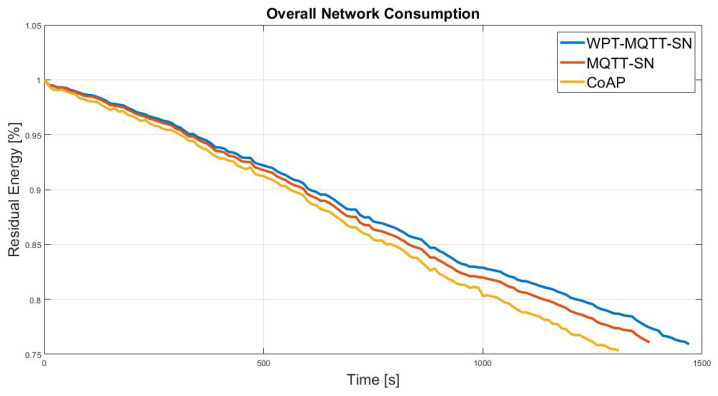
Normalised average residual battery level achieved by the CoAP, MQTT-SN and WPT-MQTT-SN protocols in a Balanced Random Mesh topology nodes deployment.

First of all, we evaluated the performance in the presence of a Random Mesh nodes deployment, which is the most common IoT scenario. In this case, a mesh topology network is originated, in which the number of hops depends on the nodes spatial distribution with a constant transmission range, as depicted in Figure 6. Figure 7 shows the benefits of the proposed system in terms of overall network lifetime. Specifically, it can be noticed an increasing gain w.r.t MQTT-SN and CoAP, approaching the maximum value of 8.4% and 11.6%.

**Figure 8:**
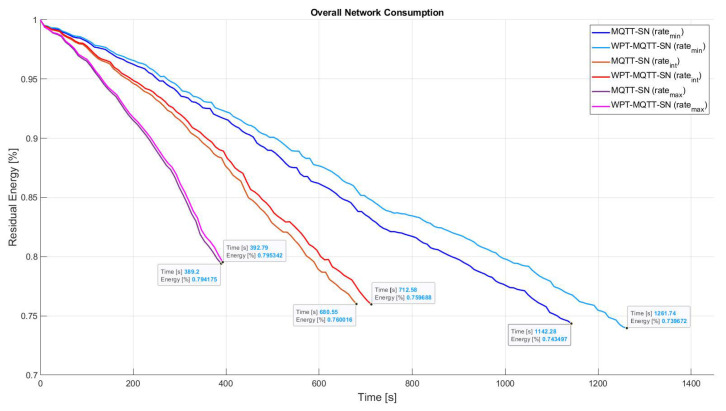
Comparison of WPT for different Publishing rates in Balanced Random Mesh topology.

Since power consumption is mainly due to packet transmission and reception, Figure 8 represents the effect of different publishing rate on the overall network lifetime. Three different publishing rates ($PR_{\max}$, $PR_{int}$, $PR_{\min}$) have been considered, where $PR_{\min}=2$ Publish pkt/s, $PR_{int}=2PR_{\min}$ and $PR_{\max}=2PR_{int}$.

We can note that the greater improvement is achieved with the lowest rate, whereas higher rates have a negligible impact on the recharging process due to the increased nodes battery consumption. The maximum achievable network lifetime gain is finally summarized in Table 2.

**Figure 9:**
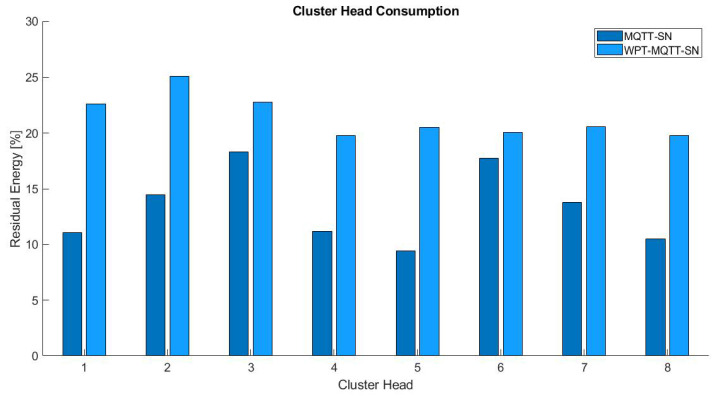
Cluster Head Energy Comparison in Balanced Random Mesh topology.

Lastly, Figure 9 shows the residual energy of every CH, which represents the WSN nodes that perform more radio transmissions and, thus, consume more energy. The average improvement of the WPT is about 37.9%, with a maximum value of 54.2%.

**Figure 10:**
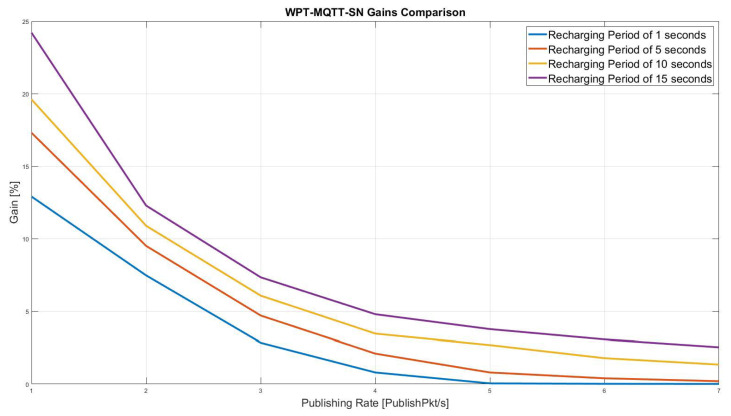
Overall Network Lifetime Gain comparison with different publishing rate and recharging period.

For the sake of comparison, Figure 10 shows the gain achieved by the proposed approach in terms of overall network lifetime, varying the recharging period of the RNs from 1 to 15 s. It can be noticed that, theWPT-MQTT-SN performance decreases at the increasing of publishing rates. However, the better result is obtained with the highest recharging period, even though this approach causes an increase of packet loss, since during the charging period, RNs cannot communicates with internal or external nodes.

**Figure 11:**
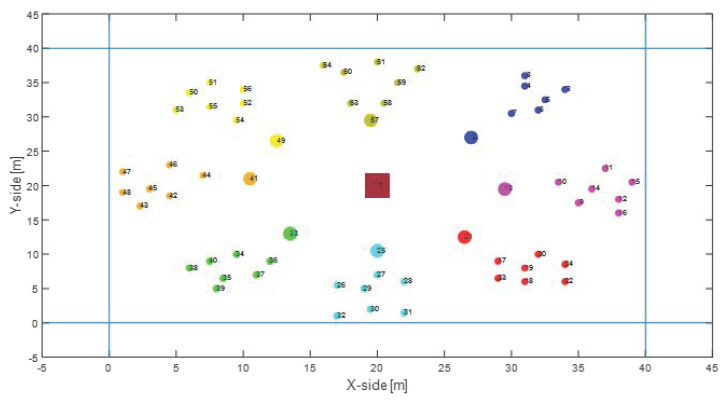
Two-Tier star-based nodes deployment, where clusters are highlighted in different colours.

An alternative scenario that has been investigated consists in a Two-Tier star-based nodes deployment, in which the ES is located at the barycenter of the network surrounded by a first tier of CHs, where all the other nodes are one-hop distant from them, as represented in Figure 11.

**Figure 12:**
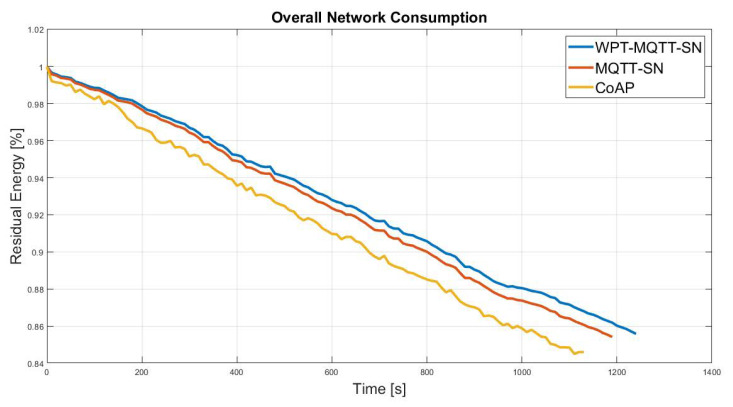
Normalised average residual battery level achieved by the CoAP, MQTT-SN and WPT-MQTT-SN protocols in a Two-Tier star based topology nodes deployment.

**Figure 13:**
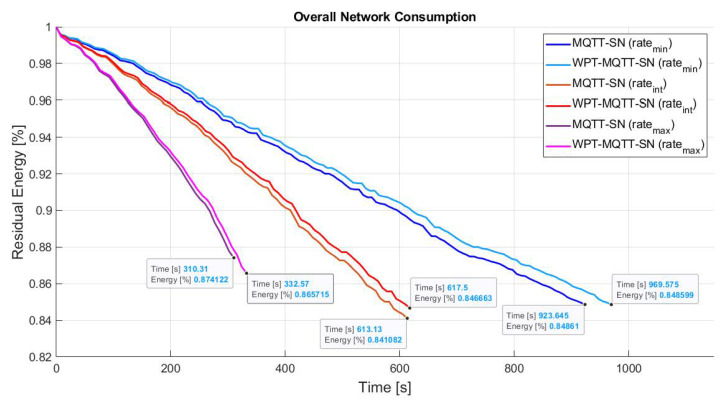
Comparison of MQTT-SN protocols without and with WPT for different Publishing rates in Two-Tier star based topology.

Simulation performed on this scenario have highlighted a reduction of the network lifetime, as depicted in Figure 12. This results depends mainly on the node spatial distribution, as nodes that represent bottleneck, i.e., CHs, are more stressed. As a matter of fact, the traffic from/to single cluster is entirely managed by its CH, without the possibility of offloading the workload according to the nodes battery level. In this case, the difference between the residual energy of the classic MQTT-SN without WPT and the proposed approach is 4.5%, while CoAP loses approximately 8.9%. Moreover, as the Random Mesh topology the performance of MQTT-SN-WPT degrades with the increasing of the publishing rate, as shown in Figure 13.

**Figure 14:**
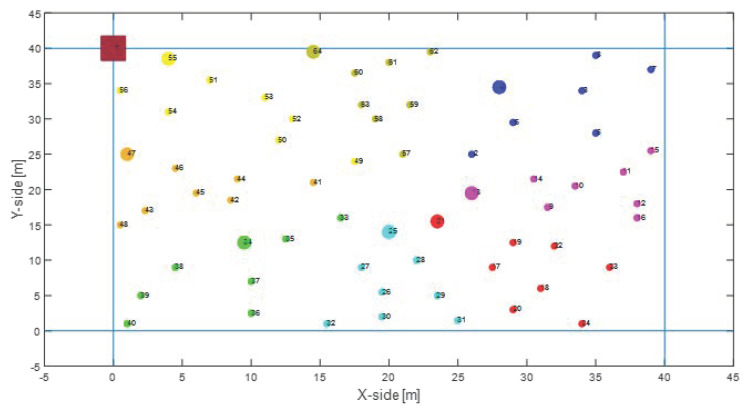
Unbalanced Random Mesh nodes deployment, where clusters are highlighted in different colours.

For the sake of completeness, the proposed solution have been tested over an Unbalanced Random Mesh nodes deployment, as shown in Figure 14, where the nodes are arranged as in Random Mesh topology, but the ES is positioned at the edge of the network.

**Figure 15:**
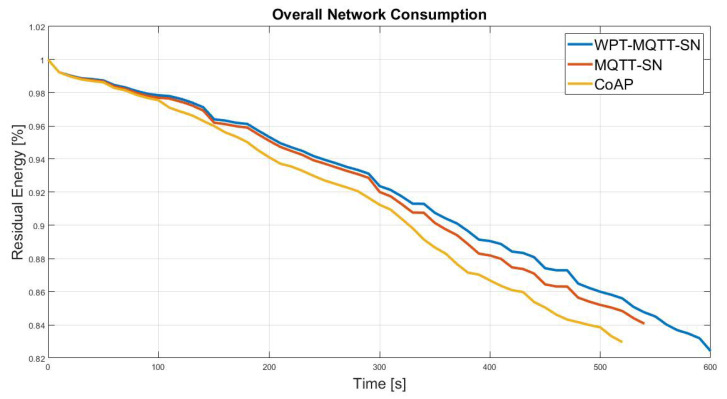
Normalised average residual battery level achieved by the CoAP, MQTT-SN and MQTT-SN-WPT protocols in a Unbalanced Random Mesh topology nodes deployment.

It is worth noticing that this represents a worst-case scenario, where performances degrades, but it is interesting to evaluate the effects of WPT. Since most of the CHs are far from the ES or are not in its coverage range, the network nodes cannot properly recharge their battery and, thus the overall network consumption of the proposed approach is similar to the MQTT-SN standard, as shown in Figure 15.

**Figure 16:**
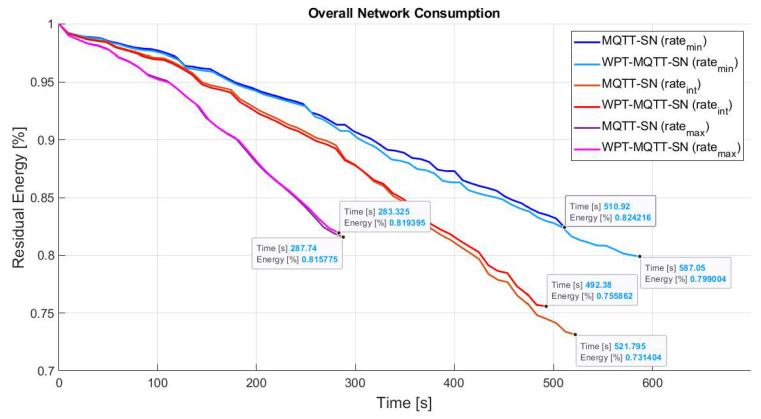
Comparison of WPT for different Publishing rates in Unbalanced Random Mesh topology.

Moreover, increasing the publishing rate the network consumption gets worse for all the solution and the effect of WPT becomes irrelevant, as depicted in Figure 16. In conclusion, the proposed WPT-MQTT-SN approaches is able to achieve remarkable efficiency in the presence of controlled topology where the ES node deployment can be spatially optimised, which represent the most common case in IWSANs.

To improve the accurateness of the results analysis, we have compared the performance achieved by the considered protocols (i.e., CoAP, MQTT-SN and WPT-MQTT-SN) over three different topologies (i.e., Random Mesh, 2-Tier and Unbalanced Random Mesh) in the same simulation time interval. The results, in terms of normalised lowest/highest residual energy and lowest/highest network lifetime are pointed out in Table 3 and Table 4, respectively, all confirming the previous considerations within a more reliable min-max confidence interval.

## 4. Conclusions

This paper deals with the design of an IoT ecosystem for industrial applications based on Web-like protocols to accommodate complex communications schemes, and to face the technological heterogeneity. In addition, the usual constraints faced by the IoT devices are considered to provide system availability and reliability, despite the limited energetic budget. Towards this goal, a general framework inspired by the integration of the Publish-Subscribe and WPT principles have been proposed, in order to jointly optimise the QoS requirements and the network lifetime. In particular, we focused on specific functionalities to handle data flows and to manage power budget scheduling to each node. The system has been accurately characterised in terms of interfaces, communications/control protocols with a specific focus on hardware architectures and components. This allowed the extension of the basic MQTT-SN protocol towards the WPT-MQTT-SN approach, that is able to optimise the energy distribution among cluster of devices, depending on the mutual interaction patterns. The integrated solution have been validated in practical scenarios and compared with existing standard, always pointing out remarkable performance in terms of network lifetime.

## Figures and Tables

**Table 1:** Parameters adopted to model a IWSAN node in simulation campaign.

	Min	Nom	Max	Unit
Supply voltage during radio operation	2.1		3.6	V
RF Frequency Range	2400		2483.5	MHz
Transmit bit	250		250	Kbps
Current consumption: Radio Trasmitting at 0 dBm		17.4		mA
Current consumption: Radio receiving		19.7		mA
Current consumptiom: Radio on, Oscillator on		365		μA
Current consumption: Idle mode, Oscillator off		20		μA
Current consumption: Sleep Mode			1	μA
Active current at Vcc = 3 V, 1 MHz		500	600	μA
Current consumption during sensing operation		15.4		mA
Voltage regulator current draw	13	20	29	μS

**Table 2:** Network lifetime gain in a Balanced Random Mesh topology for different publishing rates.

Publishing Rate	Gain [%]
Minimum	9.5
Intermediate	4.7
Maximum	0.8

**Table 3:** Normalised residual energy comparisons for different topologies and protocols.

	Random		Two-Tier		Unbalanced	
	Min	Max	Min	Max	Min	Max
**WPT-MQTT-SN**	0.78%	0.81%	0.83%	0.90%	0.81%	0.92%
**MQTT-SN**	0.75%	0.79%	0.82%	0.89%	0.79%	0.91%
**CoAP**	0.72%	0.76%	0.79%	0.85%	0.76%	0.83%

**Table 4:** Overall network lifetime comparisons for different topologies and protocols.

	Random		Two-Tier		Unbalanced	
	Min	Max	Min	Max	Min	Max
**WPT-MQTT-SN**	1043	1375	732	985	409	737
**MQTT-SN**	909	1153	664	923	381	702
**CoAP**	805	1014	596	898	305	624

## Data Availability

Not applicable.

## References

[1] Voas J. (2016). Demystifying the Internet of Things. Computer.

[2] Weyrich M., Ebert C. (2016). Reference Architectures for the Internet of Things. IEEE Softw..

[3] Wollschlaeger M., Sauter T., Jasperneite J. (2017). The Future of Industrial Communication: Automation Networks in the Era of the Internet of Things and Industry 4.0. IEEE Ind. Electron. Mag..

[4] Wang K., Wang Y., Sun Y., Guo S., Wu J. (2016). Green Industrial Internet of Things Architecture: An Energy-Efficient Perspective. IEEE Commun. Mag..

[5] Raggett D. (2015). The Web of Things: Challenges and Opportunities. Computer.

[6] Heuer J., Hund J., Pfaff O. (2015). Toward the Web of Things: Applying Web Technologies to the Physical World. Computer.

[7] Bormann C., Castellani A.P., Shelby Z. (2012). CoAP: An Application Protocol for Billions of Tiny Internet Nodes. IEEE Int. Comput..

[8] Gomez C., Arcia-Moret A., Crowcroft J. (2018). TCP in the Internet of Things: From Ostracism to Prominence. IEEE Int. Comput..

[9] Quincozes S., Emilio T., Kazienko J. (2019). MQTT Protocol: Fundamentals, Tools and Future Directions. IEEE Lat. Am. Trans..

[10] Huang J., Zhou Y., Ning Z., Gharavi H. (2019). Wireless Power Transfer and Energy Harvesting: Current Status and Future Prospects. IEEE Wirel. Commun..

[11] Alkheir A.A., Al-Anbagi I.S., Mouftah H.T. A Statistical Analysis of RF-Energy Harvesting in Wireless Networks. Proceedings of the 2015 IEEE International Conference on Ubiquitous Wireless Broadband (ICUWB).

[12] Lu J., Liu S., Wu Q., Qiu Q. Accurate modeling and prediction of energy availability in energy harvesting real-time embedded systems. Proceedings of the International Conference on Green Computing.

[13] Mirbozorgi S.A., Yeon P., Ghovanloo M. (2017). Robust Wireless Power Transmission to mm-Sized Free-Floating Distributed Implants. IEEE Trans. Biomed. Circuits Syst..

[14] Bonanni M., Chiti F., Fantacci R., Pierucci L. (2021). Dynamic Control Architecture Based on Software Defined Networking for the Internet of Things. Future Int..

[15] Chiti F., Fantacci R., Pierucci L. (2021). A Green Routing Protocol with Wireless Power Transfer for Internet of Things. J. Sens. Actuator Netw..

[16] Cidronali A., Collodi G., Maddio S., Passafiume M., Pelosi G. (2018). 2-D DoA Anchor Suitable for Indoor Positioning Systems Based on Space and Frequency Diversity for Legacy WLAN. IEEE Microw. Wirel. Components Lett..

[17] Maddio S., Cidronali A., Passafiume M., Collodi G., Lucarelli M., Maurri S. (2017). Multipath Robust Azimuthal Direction of Arrival Estimation in Dual-Band 2.45?5.2 GHz Networks. IEEE Trans. Microw. Theory Tech..

[18] Collodi G., Maddio S., Pelosi G. (2021). Design of a Compact and Highly Efficient Energy Harvester System Suitable for Battery-Less Low Cost On-Board Unit Applications. Electronics.

[19] Passafiume M., Collodi G., Cidronali A. (2020). Design Principles of Batteryless Transponder for Vehicular DSRC at 5.8 GHz. IEEE J. Radio Freq. Identif..

[20] Maddio S., Passafiume M., Cidronali A., Manes G. (2015). A Distributed Positioning System Based on a Predictive Fingerprinting Method Enabling Sub-Metric Precision in IEEE 802.11 Networks. IEEE Trans. Microw. Theory Tech..

